# Regulation of Innate Lymphoid Cells by Aryl Hydrocarbon Receptor

**DOI:** 10.3389/fimmu.2017.01909

**Published:** 2018-01-05

**Authors:** Shiyang Li, John W. Bostick, Liang Zhou

**Affiliations:** ^1^Department of Infectious Diseases and Immunology, College of Veterinary Medicine, University of Florida, Gainesville, FL, United States; ^2^Department of Chemical and Biological Engineering, Northwestern University, Evanston, IL, United States

**Keywords:** aryl hydrocarbon receptor, innate lymphoid cell, mucosal immunity, gut, microbiota and immunity

## Abstract

With striking similarity to their adaptive T helper cell counterparts, innate lymphoid cells (ILCs) represent an emerging family of cell types that express signature transcription factors, including T-bet^+^ Eomes^+^ natural killer cells, T-bet^+^ Eomes^−^ group 1 ILCs, GATA3^+^ group 2 ILCs, RORγt^+^ group 3 ILCs, and newly identified Id3^+^ regulatory ILC. ILCs are abundantly present in barrier tissues of the host (e.g., the lung, gut, and skin) at the interface of host–environment interactions. Active research has been conducted to elucidate molecular mechanisms underlying the development and function of ILCs. The aryl hydrocarbon receptor (Ahr) is a ligand-dependent transcription factor, best known to mediate the effects of xenobiotic environmental toxins and endogenous microbial and dietary metabolites. Here, we review recent progresses regarding Ahr function in ILCs. We focus on the Ahr-mediated cross talk between ILCs and other immune/non-immune cells in host tissues especially in the gut. We discuss the molecular mechanisms of the action of Ahr expression and activity in regulation of ILCs in immunity and inflammation, and the interaction between Ahr and other pathways/transcription factors in ILC development and function with their implication in disease.

## Introduction

Innate lymphoid cells (ILCs) are newly identified cell populations, which mirror helper T cells, such as Th1, Th2, and Th17 cells, by expressing similar transcription factors and cytokines (1–3). ILCs are divided into group 1 ILCs (ILC1) (T-bet^+^), group 2 ILCs (ILC2) (GATA3^+^), and group 3 ILCs (ILC3) (RORγt^+^) (1). To join the group, a new type of ILC that express the transcription factor Id3 and exhibit regulatory function [known as regulatory ILC (ILCreg)] have also recently been identified (4). Notably, natural killer (NK) cells have been defined as distinct population from ILC1, based on eomesdermin (Eomes) expression and a distinct progenitor from other ILCs (5). ILCs are predominantly locate at the mucosal barriers and participate in various biological processes, such as control of pathogenic infection, progression of autoimmune disease, as well as development of cancer (2, 6, 7). Different from adaptive immune cells, ILCs lack the antigen stimulation step and respond quickly under certain contexts of disease (8). The aryl hydrocarbon receptor (Ahr) is a ligand-dependent transcriptional factor, which can sense environmental and endogenous compounds generated by commensal, dietary, or cellular metabolism (9–11). Ahr has been studied in the development and/or function of various immune/non-immune cells (11) and recently found to be key regulator of ILC3 (12–14). There are many extensive reviews on Ahr in other immune cells. In this review, we focus our efforts on summarizing the recent progresses on decoding Ahr physiological functions in the development and function of ILCs, as well as Ahr-mediated cross talk between ILCs and other immune/non-immune cells in host tissues, especially in the gut. We discuss the molecular regulation of Ahr expression and activity in ILCs, and the interaction between Ahr and other pathways/transcription factors in ILC development and function. We also identify areas that need further study, especially the role of Ahr in group 1 and group 2 ILCs.

## Description and Function of ILCs

Innate lymphoid cells share the same progenitor, common lymphoid progenitors (CLPs), as adaptive immune cells, including T and B cells (15). CLPs differentiate toward unique direction to α-lymphoid precursor, and then common helper innate lymphoid progenitor (CHILP), to become ILCs, including NK cells, ILC1, ILC2, ILC3, and ILCreg (5, 16). The lineage-defining transcription factors, key regulators, stimuli, and effector molecules are summarized in Table 1.

**Table 1:** Features of innate lymphoid cell (ILC) subsets.

Nomenclature		Lineage-defining transcription factors	Key transcription factors	Stimuli	Effector molecules
Group 1 ILCs (ILC1)	Natural killer cells	T-bet, Eomes	ETS1, Blimp1, KLF4, Helios, TOX, Nfil3, Id2, aryl hydrocarbon receptor (Ahr)	IL-12	IFNγ, TNFα, perforin, granzymes
				IL-15	
	ILC1	T-bet	GATA3, Nfil3, Id2, Ahr	IL-18	IFNγ, TNFα

Group 2 ILCs (ILC2)	ILC2	GATA3	Gfi1, RORα, Bcl11b, TCF1, G9A, ETS1, Nfil3, Id2, Notch	IL-25	IL-4, IL-5, IL-9, IL-13, Areg
				IL-33	
				Thymic stromal lymphopoietin	
				TNF-like ligand 1A	
				IL-15	

Group 3 ILCs (ILC3)	NCR^+^CCR6^−^ ILC3	RORγt, T-bet	Ahr, WASH, GATA3, Nfil3, Id2, Ikaros, Notch	IL-23	IL-22, IFNγ, GM-CSF
		IL-1β			
	NCR^−^CCR6^+^ ILC3	RORγt	Ahr, GATA3, Nfil3, Id2, Ikaros	IL-15	IL-22, IL-17
	NCR^−^CCR6^−^ ILC3	RORγt, T-bet	Ahr, GATA3, Nfil3, Id2, Ikaros	IL-18	IL-22, IL-17, IFNγ
	Fetal lymphoid tissue inducer	RORγt	Id2, Ikaros, GATA3, Nfil3	NA	Lymphotoxin

Regulatory ILC (ILCreg)	ILCreg	Id3	Id3	TGFβ	IL-10

### Group 1 ILC

While NK cells are predominantly circulating in the blood and secondary lymphoid organs such as the lymph nodes and spleen, NK cells are also found in some non-lymphoid tissues such as the liver, uterus, and lung (17). Closely related in function to NK cells, ILC1 are present in various non-lymphoid tissues, including intestine, liver, salivary glands, and the female reproductive tract (18). The development and function of ILC1 depend on T-bet, while the requirement of T-bet by NK cells appears to be complicated since deletion of T-bet reduces the numbers of NK cells in liver, spleen, and peripheral blood (19, 20), but not in bone marrow and intestine (5, 19, 20). The transcription factor, Eomes, distinguishes NK cells from ILC1 and is indispensable for the development of NK cells (5). Recent studies indicate that NK cells and ILC1 derive from different progenitors, which further separate NK cells from ILC1 (5). Although developmentally identified as two distinct populations, NK cells and ILC1 can be stimulated by IL-12, IL-15, or IL-18 to produce interferon γ (IFNγ) and tumor necrosis factor (TNF) (18), which are critical for the immune response to control intracellular pathogens, viruses, and tumors (5, 21, 22). NK cells have the ability to secrete granzyme and perforin to promote cytotoxic function, which imparts NK cells tumor suppression activity, distinct from ILC1 (23). Different from intestinal lamina proprial ILC1 that express T-bet but not Eomes, intraepithelial ILC1 have been shown to express both T-bet and Eomes, and produce granzyme and perforin; however, lack of the requirement of IL-15 signals for their maintenance distinguishes intraepithelial ILC1 from NK cells (24).

### Group 2 ILC

Group 2 ILCs have been identified to localize in various lymphoid/non-lymphoid tissues, including intestine, lung, adipose tissue, spleen, nasal tissue, and skin, while immature ILC2 are also reported in bone marrow (25–27). The development and function of ILC2 require GATA3, RORα, Gfi1, TCF1, Bcl11b, and Notch signaling, of which GATA3 acts as the defining marker of ILC2 (26, 28–34). Upon stimulation with IL-25, IL-33, or thymic stromal lymphopoietin (TSLP), ILC2 can produce IL-5, IL-13, and IL-4, similar to Th2 cells, which contribute to the control of helminth infection and pathology of allergic inflammation (25, 35–39). ILC2 can also express IL-9 to promote the epithelial cell maintenance in the lung (40, 41). Amphiregulin is an effector molecule produced by ILC2 to participate in the tissue repair in the gut (42). Additionally, ILC2 have been shown to promote the beiging of white adipose tissue to control obesity through the production of methionine-enkephalin peptides (43, 44).

### Group 3 ILC

Group 3 ILCs are mainly found in gastrointestinal tract, while few ILC3 are present in other tissues (45, 46). ILC3 are heterogeneous, and can be divided, based on the expression of the natural cytotoxicity receptor (NCR or NKp46/NKp44) and chemokine receptor 6 (CCR6), into three major groups: NCR^+^CCR6^−^ ILC3, NCR^−^CCR6^+^ ILC3, and NCR^−^CCR6^−^ ILC3 (47). It should be noted that the above discussion is on ILC3 after birth. Fetal ILC3, also known as lymphoid tissue inducer (LTi) cells, which express RORγt, function in the formation of secondary lymphoid organs, such as lymph nodes and gut-associated lymphoid tissue (48–50). Postnatal CCR6^+^ ILC3 found in the gut and other lymphoid organs are known as LTi-like cells (51). While RORγt is the common transcription factor that is required for the development, maintenance, and function of all ILC3 (52), NCR^+^ ILC3 also appear to depend on T-bet for development and function (53). When stimulated, all three subsets of ILC3 produce IL-22, while NCR^+^ ILC3, relying on T-bet, can express IFNγ (53). In addition, ILC3 can also secret IL-17A and GM-CSF (51, 54). GATA3 is required for development of all IL-7Rα-expressing ILCs (55). Although GATA3 expression is high in ILC2, it is also expressed at a lower level in ILC1 and ILC3 and required for their maintenance (5, 56). It has been shown that GATA3 is important for ILC3 function to produce IL-22 (47). ILC3 are involved in clearance of bacterial and fungal infection, control of enteric virus infection, and maintenance of microbiota (57–62), while recent studies suggest that GM-CSF, as well as IL-22, expressed by ILC3 participate in ILC-driven colitis (63–65). After birth, ILC3 are also required for the development of cryptopatches and isolated lymphoid follicles (ILFs) in the gut through expression of lymphotoxin and CCR6 (66–69).

### Regulatory ILC

In addition to ILCs discussed above, a new ILC subset, with the ability to suppress ILC1 and ILC3 to promote the resolution of intestinal inflammation, has been identified recently in mice (4). Although further work is needed to confirm the existence and function of this cell type, ILCreg, mainly populate in the gut, develop from CHILP in bone marrow, and require transcription factor Id3 for their development. The regulatory function of ILCreg is mediated by IL-10. TGFβ1 is required for the expansion of ILCreg during inflammation (4). In human, the regulatory ILC (ILCreg) are also reported in the context of cancer recently (70), to suppress the expansion of tumor-associated T cells. Different from the mouse ILCreg that do not express other ILC signature transcription factors, the human ILCreg, present in the tumor tissue, express high levels of Eomes, T-bet, GATA3, RORα, and Ahr, suggesting an overlapping transcriptional profile of the human ILCreg and other ILC subsets.

## Ahr Structure and Activation

Aryl hydrocarbon receptor is a ligand-dependent transcription factor and belongs to Per-Arnt-Sim (PAS) superfamily (71, 72). Various Ahr ligands have been identified, including environmental pollutants such as dioxins, and multiple physiologic ligands generated by microbiota, diet, and host metabolism (73–76). Without ligand binding, Ahr localizes in the cytoplasm, and this inactive status is maintained by interacting with 90-kDa heat shock protein (HSP90) (77). Ahr also interacts with Ahr-interacting protein (AIP) which protects Ahr from degradation (78), as well as p23 (79). Upon ligand activation, the conformation of Ahr is changed, leading to the release of Ahr from the protein complex and the translocation of Ahr into the nucleus, where Ahr interacts with Ahr nuclear translocator (ARNT) through PAS-A domain and bHLH domain (80) and acts as a transcription factor targeting dioxin response element (DRE)-containing genes, which are prototypically cytochrome P450 family, like Cyp1a1, but also include genes involved in other important biological processes (13, 81). Several partners of Ahr have been identified, such as RORγt, sterol regulatory element binding transcription factor 1, LXR, NF-κB (13, 82, 83). The involvement of ARNT in these reported interactions remains to be determined.

Aryl hydrocarbon receptor was initially identified as the sensor for 2,3,7,8-tetracholrodibenzo-p-dioxin (TCDD) (84). Later, a variety of Ahr ligands were identified from different physiological sources, such as tryptophan (Trp) metabolism and microbiota. The metabolism of Trp generates Ahr ligands through catalysis by indoleamine-2,3-dioxygenase (IDO) and tryptophan-2,3-dioxygenase (TDO) to kynurenine (Kyn), which acts as an Ahr ligand (76, 85, 86). Independent of IDO/TDO, Trp can also be metabolized by the tryptamine and serotonin pathway, of which the metabolites can act as Ahr agonist (87, 88). Notably, Trp can be photo-oxidized by ultraviolet light or metabolized by other pathways to 6-formylindolo[3,2-b]carbazole (FICZ), which has been proven as a physiologically relevant Ahr agonist (89, 90). Of note, a higher concentration of Kyn, at micromolar concentration, compared to nanomolar of TCDD or FICZ, is required for Ahr activation.

In addition to cellular metabolism, commensal bacteria can catalyze Trp into Ahr ligands as well (74, 91). Lactobacilli expand when the energy source switches from sugar to Trp, and produce indole-3-aldehyde which acts as Ahr ligand to promote IL-22 production by ILC3 (74). Consequently, the Ahr-IL-22 axis provides resistance to the fungus *Candida albicans* and protection from dextran sulfate sodium (DSS)-induced colitis. In accordance with the importance of Trp in mice, recent research suggests that dysregulation of commensal bacteria that use Trp to generate Ahr ligands may correlate with the pathogenesis of human inflammatory bowel disease (IBD) (92). Besides the Ahr ligands generated by cellular metabolism or commensal bacteria, bacterial pigment factors such as the phenazines from *Pseudomonas aeruginosa* and the naphthoquinone phthiocol from *Mycobacterium tuberculosis* can also act as ligands for Ahr, and contribute to the antibacterial response through activation of the Ahr pathway (93).

## Ahr Expression in ILCs

Aryl hydrocarbon receptor is thought to be expressed ubiquitously in various organs and cell types, including immune cells, such as Th17 cells, IL-17-producing γδ T cells, Treg cells, CD8αα IEL lymphocytes, B cells, Langerhans cells, monocytes, and splenic dendritic cells (DCs) (94–100). However, the expression of Ahr in ILCs, at both mRNA and protein level, remains to be clarified. Genome-wide transcription analysis of different ILC populations, which is available at IMMGEN.ORG, has shown that *Ahr* mRNA is detectable among ILCs (101). It has been reported that cytokine stimulation, including IL-2, IL-12, or IL-15, can enhance Ahr expression in splenic NK cells (102, 103). In addition, the transcription factor, Distal-Less Homeobox 3 is found to enhance Ahr transcription in NK cells, while its function remains to be determined (104).

We and other groups have reported the expression of Ahr in ILC3. Differential levels of Ahr were observed in different subsets of ILC3 (13, 37, 41). NCR^+^ ILC3 express higher Ahr than the other two subsets of ILC3, which lack NCR on the surface (13). How Ahr expression is regulated in ILCs has been a subject of active research. Recent study has shown that in NCR^+^ ILC3, Wiskott-Aldrich syndrome protein and SCAR homolog (WASH) activates Ahr expression by recruiting AT-Rich Interaction Domain 1A (Arid1a) to the *Ahr* promoter, and thus maintains NCR^+^ ILC3 in the gut (105).

Although further investigation on Ahr expression, especially at the protein level, needs to be conducted, the public data at IMMGEN.ORG appears to show that the special microenvironment of the gut correlates with the high Ahr transcriptional expression, since lower Ahr expression is observed in spleen or liver NK cells or ILC1. In a *Cyp1a1* (a target gene of Ahr) reporter mouse, Ahr was shown mainly active in the gut in homeostatic conditions (106). A recent paper using a mouse model in which GFP was knocked into the endogenous locus of Ahr showed that among Tregs in various tissues, gut Treg cells express the highest amounts of Ahr, suggesting a tissue adaptation of Ahr expression (107). Identification of the gut specific factors, such as cytokines/metabolites and transcription factors that facilitate Ahr expression will provide insights into the regulation of Ahr expression in ILCs, and potentially be translated into clinical manipulation of the Ahr pathway. To get a molecular understanding on the regulation of Ahr expression, it is of importance to analyze chromatin status of the Ahr locus and Ahr interactions with key transcription factors in different ILC populations.

## Involvement of Ahr in ILC Function and Regulation

### Ahr and NK Cells/ILC1

In tumor, Ahr promotes NK cell cytotoxicity and its production of IFNγ (103). During *T. gondii* infection, Ahr is also required for maximal IL-10 production by NK cells (102). It has also been shown that Ahr maintains liver-resident CD49a^+^ cells by regulating cytokine-induced cell death (108). Notably, CD49a is considered as a marker for ILC1 in the liver, instead of NK cells (18). Therefore, these data may suggest that Ahr is required for liver ILC1 maintenance (108).

So far, the studies on Ahr in NK cells or ILC1 have been predominantly focused in the liver or spleen. The function of Ahr in the gut ILC1 and NK cells still remains to be elucidated, given that the gastrointestinal tract is another site for these two cell populations, especially for ILC1 (5).

### Ahr and ILC2

Currently, limited knowledge is available on the function of Ahr in ILC2. IFNγ has recently been shown to inhibit ILC2 activation (109, 110). In addition, IFNγ can induce *Ido1* mRNA and Ido protein expression in some cell types (111, 112). Given that Ido1 is able to catalyze Trp to Kyn, which acts as a ligand for Ahr, it is tempting to speculate that Ahr ligands, such as Kyn, might suppress ILC2 function but additional works are needed to test this hypothesis. TNF-like ligand 1A (TL1A) has been shown to promote expansion and function of ILC2 in the gut (113). RNA-seq data reveal that TL1A enhances Ahr expression in the presence of IL-33 and IL-25 in human ILC2 (114). Thus, the function of Ahr in ILC2 and in *in vivo* models of ILC2-driven pathology remains to be investigated.

### Ahr and ILC3

**Figure 1:**
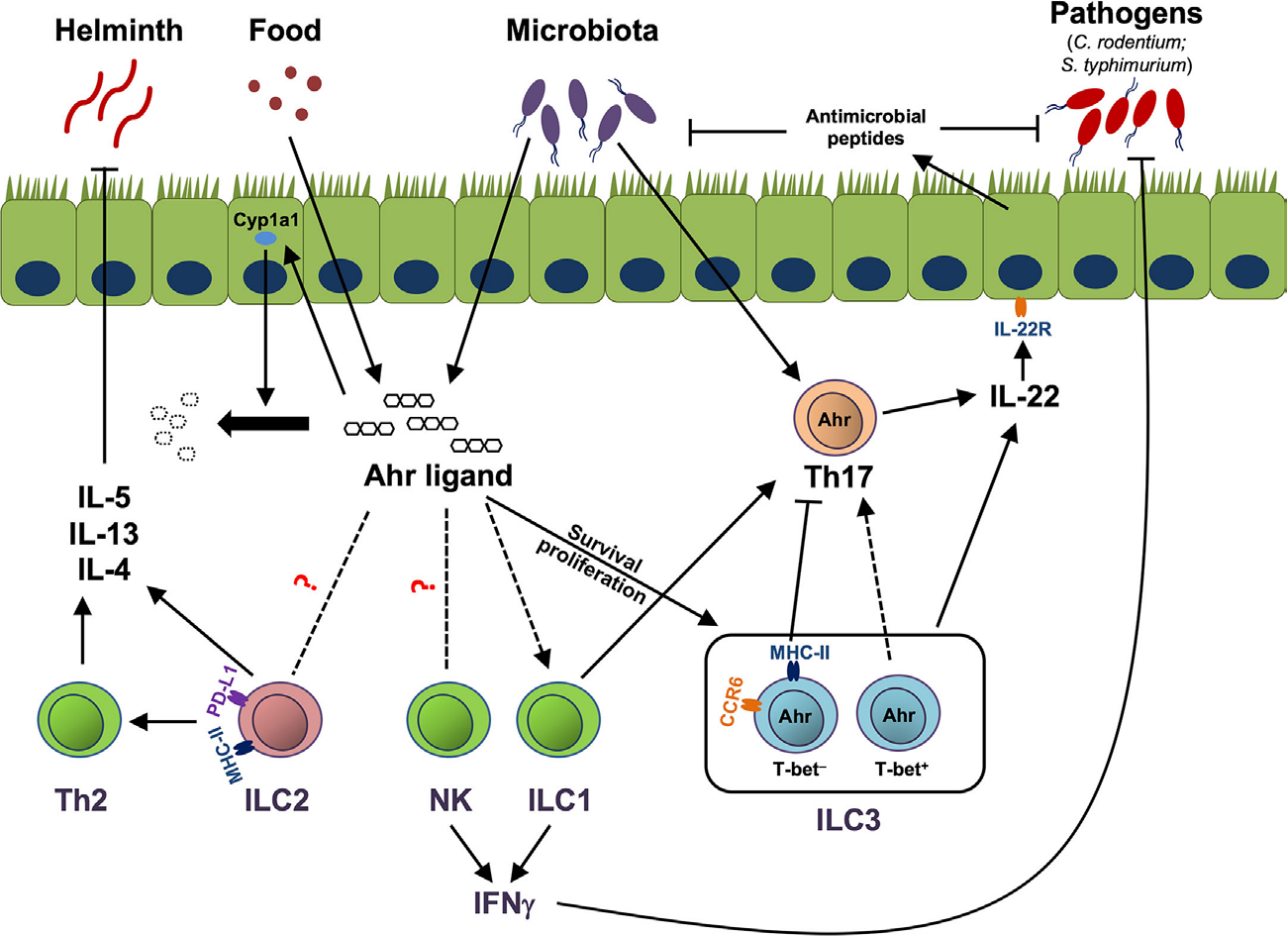
Aryl hydrocarbon receptor (Ahr)-mediated cross talk between innate lymphoid cells (ILCs) and immune/non-immune cells. Ahr ligands derived from the diet or microbiota activate Ahr to promote group 3 ILCs (ILC3) homeostasis by enhancing the survival or proliferation of ILC3. MHC-II^+^ ILC3, which are mainly CCR6^+^, suppress pathogenic Th17 response to commensal bacteria, while T-bet^+^ ILC3, together with group 1 ILC (ILC1), promote Th17 cells. Both Th17 cells and ILC3 can produce IL-22 to control commensal/pathogenic bacteria through facilitating the production of antimicrobial peptides by epithelial cells. Ahr ligand could potentially regulate natural killer (NK) cells, ILC1, and group 2 ILCs (ILC2) through the Ahr pathway in the gut. NK cells and ILC1 can help the host to clear pathogens, like *Salmonella typhimurium*, by production of effector cytokine IFNγ. ILC2, through expression of MHC class II (MHC-II) and programmed death ligand 1 (PD-L1), enhance Th2 cells. ILC2 and Th2 cells protect the host from helminth infection by secreting type 2 cytokines, including IL-5, IL-13, and IL-4. Ahr ligand enhances Cyp1a1 expression in gut epithelial cells, and as a feedback negative control loop, Cyp1a1 degrades Ahr ligand to prevent overt Ahr-mediated immune responses. Solid lines and arrows depict known regulation. Dotted lines and arrows depict to-be-determined regulation in the gut.

Aryl hydrocarbon receptor has been relatively well studied in gut ILC3. Although it is dispensable for fetal LTi development, Ahr is essential for the maintenance and IL-22 production of ILC3 (12, 13, 45). Although the precise mechanisms by which Ahr regulates the homeostasis of ILC3 still remain to be determined, it has been described that Ahr can regulate survival and/or proliferation of ILC3 (Figure 1). First, it is reported that Ahr is important for the survival of ILC3 by promoting the expression of anti-apoptotic proteins, such as Bcl-2. Ahr upregulates IL-7 receptor (IL-7R) in ILC3, in line with the role of IL-7/IL-7R signaling pathway in the supporting the survival of ILC3 (13). Second, it has been shown that Ahr-deficient ILC3 have reduced Ki67 expression, indicating that decreased proliferation may lead to the defective expansion of ILC3. Furthermore, Ahr can regulate the expression of Kit through binding to DRE at the promoter of *Kit* locus, suggesting direct regulation of Kit expression by Ahr at the transcriptional level (12). Finally, Ahr supports the development of ILC3 presumably through promoting the transcription of Notch 1 and Notch 2, although defects in Notch signaling have more effect on NCR-expressing ILC3 than NCR-negative ILC3 (45). By regulating the maintenance and function of ILC3, Ahr is critical for the clearance of *Citrobacter rodentium*, a murine pathogen that models human enterohemorrhagic *Escherichia coli* and enteropathogenic *E. coli* infections in the gut (12, 13, 64), as well as for the pathology of anti-CD40-incuced colitis (64).

### Ahr in ILC Plasticity

The plasticity of ILCs has been observed in both human and mouse systems under steady state or certain disease models, while the mechanism that drives the plasticity of ILCs is still not well understood. The conversion of ILC3 to ILC1 is characterized by the loss of RORγt and gain of T-bet expression to become exILC3 (115, 116). These exILC3 stop the production of IL-22, and begin to secrete IFNγ. IL-15 and IL-12 can lead to downregulation of RORγt, and enhance IFNγ expression (116). In support, IL-12 has been shown to participate in the transition of ILC3 to ILC1 in humans (115, 117). There is an increase of ILC1 and decrease in ILC3 in the intestines of patients with Crohn’s disease, suggesting the ILC3-derived ILC1 might contribute to the pathology of human IBD (115, 117). Human ILC1 can also convert to ILC3 in the presence of IL-2, IL-23, and IL-1β, and retinoic acid can accelerate this process which may depend on the receptors for retinoic acid (117). Although Ahr has been shown to prevent the differentiation of human ILC3 to NK cells (118), it is of interest to determine whether Ahr participates in the transition of ILC3 to ILC1. Of note, microbiota has been shown to maintain the RORγt expression by ILC3 through IL-7 signaling in the gut (116). Since commensal bacteria have the ability to produce Ahr ligands, Ahr might receive the signals from microbiota to maintain ILC3 through upregulating IL-7R.

The plasticity of ILC2 has recently been reported (31, 119–121). Gfi1, a key transcription factor for ILC2 development and function, appears to sustain ILC2, as deletion of Gfi1 in ILC2 leads to upregulation of RORγt and IL-17 production by these ILC2 (29). Similarly, Bcl11b, a recently defined transcription factor for ILC2, maintains the stability of ILC2 by suppressing RORγt and Ahr expression (31). ILC2 were found to convert to IFNγ-producing ILC1 in the lung by IL-12 and IL-18 (119–121). The conversion is dependent on T-bet expression, and enhanced by IL-1β through induction of the IL-12 receptor alpha (Il12rα). As the frequency of ILC1 shows positive correlation with disease severity in patients with chronic obstructive pulmonary disease or chronic rhinosinusitis with nasal polyps (119, 121), the plasticity of ILC2 could be a therapeutic target for these respiratory diseases. Whether Ahr plays any role in ILC2 conversion to ILC1 needs to be established.

## Ahr-Mediated Modulation of the Cross Talk Between ILCs and Other Cells

### Cross Talk with Innate Immune Cells

Innate immune cells, such as dendritic cells (DCs) and mononuclear phagocytes (MNPs), have been shown to interact with ILCs. Both CX3CR1^+^ MNPs and CD103^+^ DCs can induce IL-22 production by ILC3, while CX3CR1^+^ MNPs can also recruit ILC3 to the gut through CXCL16–CXCR6 pathway (122–124). Ahr controls the differentiation and function of DCs by arresting the differentiation of progenitors, as well as regulating antigen presentation in DCs (125–128). In addition, recent work has shown that Ahr controls differentiation of monocyte to monocyte-derived macrophages in the human system (129). Thus, it is possible that Ahr controls ILC3 through regulating these innate immune cells. On the other hand, innate immune cells can be attracted by ILC-secreted cytokines. IL-5 and IL-13, produced by ILC2, can recruit eosinophils which are essential for the clearance of helminth infections (25). ILC3 can secrete IL-17A, which is proved to attract neutrophils into the intestine (130, 131). Thus, lack of ILC3 in Ahr-deficient mice may account for the resistance of anti-CD40 colitis (64).

### Cross Talk with Adaptive Immune Cells

The absence of Ahr in ILC3 leads to defects in the IL-22-producing ability of ILC3. The impaired IL-22 production in the gut of Ahr-deficient mice causes a decrease in antimicrobial peptide production by gut epithelial cells (62, 132), leading to increased segmented filamentous bacteria (SFB) which has been established to induce Th17 cells in the gut (133, 134). However, recent papers also show that SFB induced IL-22 production by ILC3 can induce epithelial production of Serum Amyloid A, which in turn promotes Th17 cells (135, 136). Thus, the role of ILC3-derived IL-22 in regulating Th17 cells will require further investigation into the underlying molecular mechanisms, which are most likely indirect given the lack of expression IL-22R by immune cells. By supporting ILC3 homeostasis, Ahr controls cryptopatches formation, and consequently the genesis of ILFs in the gut (12, 137). As ILFs have been recognized as a site for the production of intestinal IgA responses (138), it is possible that Ahr contributes to B cell responses *via* the regulation of ILC3, in addition to its B cell-intrinsic roles (96, 97, 139).

Recent research showed that ILC2 are critical for memory Th2 cell responses, as impaired Th2 cells are found in sensitized mice, which lack ILC2 (140). During helminth infection, ILC2 have been shown to express the checkpoint molecule Programmed Death Ligand 1, through which ILC2 support Th2 polarization, and effective Th2 dependent-anti-helminth response (141). Additionally, ILC2, through producing IL-9, can sustain the proliferation of ILC2 and activation of Treg cells in arthritis, by which promote the resolution of inflammation (142). It is of interest to note that, with the resistance to IL-7-induced downregulation of IL-7R, ILCs limit the availability of IL-7 for T cells, thus controlling the homeostasis of T cells (143). Given that Ahr deficiency leads to reduction of ILC3, it remains to be determined whether enhanced T cell proliferation and Th17 cell differentiation observed in Ahr knockout mice are caused by increased IL-7 that is made available to T cells.

In addition to the cross talk between ILCs and adaptive immune cells through cytokines, ILCs interact with T cells through the expression of MHC class II (MHC-II) molecules on the surface. The MHC-II-mediated interaction between ILCs and T cells controls the activation or anergy of T cells (Figure 1). For example, ILC2, *via* MHC-II and co-stimulatory molecules, CD80 and CD86, interact with and activate T cells (144). Different from ILC2, ILC3 expressing MHC-II but not the co-stimulatory molecules CD80 and CD86, induce T cell apoptosis and tolerance in the gut (145, 146). However, ILC3 express CD30 ligand and OX40 ligand, which may contribute to the maintenance of CD4^+^ T cell memory (147). Although there is no direct evidence indicating whether Ahr regulates MHC-II or co-stimulatory molecule expression by ILC2 and ILC3, Ahr may mediate the cross talk between ILCs and T cells, at least through regulating ILC numbers (Figure 1). A recent study reveals that NCR-expressing ILCs, including ILC1 and NCR^+^ ILC3, support Th17 cells in inflamed central nervous system (148), which raises intriguing questions that whether similar event is evident in the gut, and how the host keeps the balance between the induction of Th17 cells by NCR^+^ ILCs, and the inhibition of Th17 cells by CCR6^+^ ILC3 through MHC-II expression (Figure 1).

### Cross Talk with Epithelial Cells

The cross talk between gut epithelial cells and ILC3 has been recently investigated. Over-expression of Cyp1a1, a target gene of Ahr, in epithelial cells consumes Ahr ligands in the gut, which consequently leads to the decrease of gut ILC3 (106) (Figure 1). These findings raise the possibility that activation of Ahr may not only promote gut ILC3 in a cell-intrinsic manner, but also maintain the ILC3 at a physiological level through controlling the availability of Ahr ligands in the gut. On the other hand, ILC3, *via* expression of IL-22 and lymphotoxin, regulate the fucosylation of epithelial cells which is critical for the host to control *Salmonella typhimurium* infection (149). In addition, ILC3, *via* producing IL-22, promote the expansion of intestinal stem cell, and consequently promote the regeneration of intestinal epithelium after gut injury (150, 151).

### Cross Talk with Commensals

Aryl hydrocarbon receptor appears to mediate the interaction of ILC3 and microbiota. The absence of caspase recruitment domain family member 9 (CARD9) results in alteration of microbiota, and the altered microbiota fail to metabolize Trp into Ahr ligands, leading to decreased ILC3 and IL-22 production, and increased susceptibility of the host to colitis (92). Accordingly, Ahr ligands are found decreased in the microbiota of IBD patients, especially in the individuals with IBD-associated single-nucleotide polymorphism within *CARD9* (rs10781499), suggesting microbiota–Ahr ligand axis may be a therapeutic target of colitis in humans (92). Although the cross talk between ILCs and microbiota remains to be further explored, genome-wide analysis at the transcriptional level of ILCs has been conducted using RNA-seq by comparing specific pathogen-free mice to those with microbiota depletion (152). Marked numbers of transcripts change significantly in all ILCs upon antibiotics treatment, but the expression profile is generally maintained. Intriguingly, depletion of microbiota shows more effects on the gene expression of ILC1 and ILC2 than that of ILC3. Given the important role of Ahr in ILC3 and Ahr could sense ligands generated by commensals, for example, *Lactobacillus reuteri* (74, 91), these findings may suggest ligands from other sources (e.g., diet) could activate the Ahr pathway in the absence of microbiota.

### Regulation of ILCs by ILCreg

With the minimal Ahr expression in mouse ILCreg at least under the steady state (4), it remains to be determined whether Ahr plays a role in ILCreg. In contrast to the mouse ILCreg, human ILCreg in cancer that suppress T cell expansion appear to express high level of Ahr, indicating potential role of Ahr in this population (70). The mouse ILCreg have been shown to regulate ILC1 and ILC3 (4), it is unclear whether ILCreg can suppress ILC2.

### ILC-Nervous System Interaction

The nervous system has been shown to affect ILCs. Glial cells in the gut, through secreting neurotrophic factors that bind to the neuroregulatory receptor rearranged during transfection (RET) on ILC3, promote the expression of IL-22, and consequently decrease the susceptibility to intestinal inflammation and infection (153). Recent studies demonstrate that among various hematopoietic cells, ILC2 uniquely express the neuropeptide neuromedin U (NMU) receptor 1 (NMUR1), which makes them respond to NMU (154–156). The activation of ILC2 by NMU leads to enhanced cell expansion and type 2 cytokine production, which promote the clearance of helminth in the gut. It remains to be determined that whether Ahr modulates ILC responses to neuromediators.

### Cooperation of Ahr and Partners in Regulating ILCs

Aryl hydrocarbon receptor has been studied for decades, and some interacting proteins, like HSP90 and AIP, have been well documented. However, only a few partners of Ahr have been functionally implicated in ILCs. In Th17 and IL-17-producing γδ T cells, Ahr regulates IL-22 expression while the molecular mechanism of action of Ahr is unclear (94, 95). However, Ahr has been shown to interact with RORγt in an overexpression system to promote IL-22 expression (13). RORγt is required for the recruitment of Ahr to the *Il22* locus, as Ahr alone fails to bind to the *Il22* locus. In contrast to the *Il22* locus, Ahr is recruited to the *Cyp1a1* locus independent of RORγt. These data raise a question regarding how Ahr, by cooperating with other transcription factors (e.g., RORγt), regulates gene expression in ILC3 and other lymphocytes (e.g., Th17 and γδ T cells). In addition to RORγt, the C2H2 zinc finger transcription factor Ikaros, a key regulator of hematopoiesis, is a binding protein of Ahr in ILC3 (157). Ikaros negatively regulates ILC3 through zinc finger 4-dependent inhibition of transcriptional activity of the Ahr by disruption of the Ahr–ARNT complex. It will be of interest to investigate whether Ikaros participates in a complex of Ahr and RORγt to regulate RORγt activity in ILC3 development and/or function. Intriguingly, Ikaros but not Ahr is required for fetal LTi cell development, demonstrating the distinct transcriptional regulation of fetal and postnatal ILC3.

As ILC3 resemble Th17 cells in regards to key transcription factor and cytokines, knowledge of the function of Ahr in Th17 cells might be adopted into ILC3 potentially. Transcription factor Musculoaponeurotic Fibrosarcoma (MAF) has been shown to be induced by TGFβ in Th17 cells to promote IL-17 production and suppress IL-22 secretion (158). Although the interaction between Ahr and MAF has been only implicated in type 1 regulatory T cells (159), the cross talk of these two proteins may provide insight into the molecular regulation of IL-22 expression in ILC3.

Aryl hydrocarbon receptor has been shown to interact with RelB, a key component of NF-κB signaling, and synergize to induce the transcription of certain genes, such as IL-6 and IL-8 in DC or macrophage (160, 161). Additionally, another component of NF-κB, RelA, binds to Ahr, and the interaction consequently promotes IL-6 transcription (162). Therefore, the interplay between Ahr and NF-κB pathway might be important for ILCs since the critical function of NF-κB has been investigated throughout various cell types.

Not limited to transcriptional function, Ahr has been reported to participate in posttranslational regulation in non-immune cells. It is described that Ahr acts as a component of cullin 4B ubiquitin ligase complex, which targets sex steroid receptors for degradation (163, 164). More investigation directed to confirm and extend this non-genomic function of Ahr in ILC and other cell types will be necessary to understand how Ahr is linked to protein degradation in different contexts.

In non-immune cells, Ahr exhibits a rhythmic expression, and its sensitivity to Ahr ligands is time-dependent (165). Reciprocally, genes associated with circadian clock and the behavioral responses of mice to circadian clock are regulated by Ahr (165). Ahr has been shown to interact with Bmal1, which forms a complex with Clock to facilitate the transcription of circadian genes (166–168). ILC2 activation and consequent eosinophil recruitment is responsive to the circadian clock, suggesting a conserved circadian mechanism in ILCs (25). Understanding of the synergetic function of Ahr and circadian signaling could improve our understanding of the basic biology of ILCs, and provide new targets of interest for regulation of ILCs.

## Translational Potential of Ahr in ILCs

Changes in ILCs have been reported in the patients with IBD. IL-22-producing ILC3 decreased in the intestine of Crohn’s patients (115, 169, 170), in line with the protective role of IL-22 on the integrity of gut barrier which has been implicated several mouse models (171). Other studies also reveal that IL-22 produced by ILC3 increased in inflammatory sites of the colons in both CD and UC patients (122, 172), which might be due to a compensatory response of the host to inflammation but also might reveal the pathological aspects of ILC3, especially NCR^+^ ILC3 (63, 64). The MHC-II expression on ILC3 is critical to induce T cell tolerance to gut commensal bacteria and avoid overt inflammation. It has been shown that pediatric IBD patients have reduced MHC-II expression on colonic ILC3, consistent with the model that compromised ILC3 regulatory function can lead to T cell-mediated inflammation (146). It has been shown that the expression of Ahr is reduced in the gut tissues from IBD patients compared to healthy controls (173). Accordingly, treatment of Ahr ligand ameliorated the pathology of several mouse colitis models, including 2,4,6-trinitrobenzenesulfonic acid (TNBS)-, DSS-, and T cell transfer-induced colitis, in which IL-22 is required (173, 174). Considering the role of Ahr in the maintenance of gut ILC3 and IL-22 production by ILC3, Ahr pathway could be potentially manipulated to regulate gut inflammation by increasing ILC3 in the gut of IBD patients. However, given the different functions between NCR^+^ ILC3 and NCR^−^ ILC3, special considerations are needed while targeting the Ahr pathway in IBD.

Type 2 immunity has been considered to mediate ulcerative colitis in human, which has been modeled by oxazolone-induced colitis in mice (175). A known Ahr ligand, 3,3’-Diindolylmethane, has been found to alleviate oxazolone-induced colitis, probably through inhibition of Th2/Th17 cells and induction of Treg cells (176). Since ILC2 express large amounts of type 2 cytokines, this population could potentially play a pathogenic role in ulcerative colitis (177). Despite the reduced expression of Ahr in IBD, the role of Ahr in ILC2 and disease pathogenesis remains to be determined. In addition, it will be of interest to investigate the balance between ILC2 (or type 2 immunity) and ILC3 in colitis. IL-33, a cytokine that acts on ILC2 and Th2 to promote the cytokine production, increased in IBD patients and in experimental colitis models of mice, including TNBS and DSS model (178). Ablation of IL-33-ST2 pathway relieves experimental colitis in mice. Of note, IL-33 and soluble ST2 have been shown increased in the colons of IBD patients (179), in line with the proinflammatory role of type 2 immunity. Thus, the functions of ILC2 and ILC3 in colitis could be dissected into two phases as ILC2 initiate the pathology *via* IL-13 (177), while ILC3, probably through IL-22, facilitate the tissue repair in the later phase of disease. However, IL-22 could also participate in the gut inflammation, highlighting its “double-edged sword” nature (65, 180). A recent study reveals that IL-33 stimulates ILC2 to secrete amphiregulin to promote tissue repair in experimental colitis (42), suggesting ILC2 at different stage of the disease and/or some subset of ILC2 (i.e., amphiregulin^+^ ILC2) may have protective function in the resolution of colitis as well.

Allergic asthma is a chronic inflammatory disease, in which type 2 cytokines, IL-4, IL-5, and IL-13 are associated with the pathology (181). These type 2 cytokines are required for IgE response, recruitment of eosinophils, and mucus production. ILC2 have been implicated in asthma, since ILC2 produce large amounts of IL-5 and IL-13, as well as IL-4 under certain context, in response to IL-33, IL-25, and TSLP (182). Additionally, recent study showed that ILC2 increase in the airways of severe asthma patients, suggesting ILC2 may contribute to airway inflammation in mouse and human (183). Although the function of Ahr in ILC2 remains to be determined, several Ahr ligands have been reported to suppress allergic airway inflammation in different mouse models, through suppressing type 2 cytokines, IL-4 and IL-5, production, eosinophilia, and specific IgE expression (184–186). Thus, study of the role of Ahr in ILC2 would provide another potential target for clinical intervention in airway inflammation, like asthma. Although type 2 cytokines have been well documented in asthma, elevated IL-17 has been noticed clinically (187). Given that IL-25 can induce a population of lung ILC2 with IL-17-producing ability, the potential role of this special ILC2 subset in the pathology of asthma in humans needs to be studied in the future.

Both pro- and antitumor action of Ahr has been implicated (188), and the potential function of Ahr in ILC-mediated tumor immunology remains largely unknown. Ahr has been demonstrated to promote the antitumor activity of NK cells (103). IL-22, mainly produced by ILC3 under the steady state, has been shown to associate with increased risk in colon cancer (189). Accordingly, IL-22-producing ILC3 are found to promote an experimental cancer model in mice (190). Therefore, understanding of the precise function of Ahr in ILCs in cancer needs to be carefully studied.

## Concluding Remarks

The tissue microenvironment may be involved in regulating the differentiation, homeostasis, and function of ILCs. Thus, the expression and activity of Ahr in ILCs from different organs under the steady state need to be carefully considered when designing therapeutics to target Ahr. Furthermore, it will be of great interest to investigate whether the Ahr level/activity in ILCs can be changed under different contexts, like in infection, inflammation, and/or cancers.

Cell-intrinsic role of Ahr in ILCs has to be determined given the broad expression of Ahr in other cell types. The molecular mechanism by which Ahr regulates the development or homeostasis of ILCs remains to be explored. Mechanistic insights of Ahr expression and/or activity in various ILC subsets or any given ILCs in different tissues are important for designing targeted strategy to modulate the Ahr function pharmacologically. It is of interest to investigate whether various ILCs have different sensitivity to Ahr ligand, or unique machinery to uptake Ahr ligand. Furthermore, single cell-omics studies involving RNA-seq and ATAC-seq analyses, together with ChIP-seq analysis of Ahr, will delineate the functional pattern and role of Ahr in regulating transcriptional landscape of ILCs. Identification of Ahr-binding partners in ILCs will provide insights into the mechanism by which Ahr cooperates with other factors to differentially regulate gene expression. These molecular findings could uncover more specific and effective therapeutic targets on the Ahr pathway, in cell-type/tissue-specific manner, in disease treatment and prevention.

## Author Contributions

SL wrote the manuscript with JB’s contribution. LZ supervised the research and edited the manuscript.

## Conflict of Interest Statement

The authors declare that the research was conducted in the absence of any commercial or financial relationships that could be construed as a potential conflict of interest. The reviewer MC and handling editor declared their shared affiliation.
